# Expectations and concerns of primary healthcare patients in rural areas and small towns in Poland regarding artificial intelligence

**DOI:** 10.1038/s41598-026-37779-2

**Published:** 2026-02-03

**Authors:** Justyna Kęczkowska, Małgorzata Płaza, Gabriela Henrykowska

**Affiliations:** 1 Kielce University of Technology, Kielce, Poland; 2 Department of Epidemiology and Public Health, Medical University of Lodz, ul. Żeligowskiego 7/9, 90-752 Lodz, Poland

**Keywords:** Health care, Medical research

## Abstract

The integration of artificial intelligence (AI) into healthcare presents transformative opportunities, but patient perspectives, particularly from digitally excluded populations, remain underexplored. This study aimed to analyze the awareness, acceptance and concerns regarding the use of AI in healthcare among primary care patients in rural and small-town regions of Poland. This is characteristic of the country, as over 60% of its population lives in such regions. It also sought to identify the demographic and psychosocial determinants of trust in AI. A cross-sectional survey was conducted using a paper questionnaire distributed to 545 adult patients in three primary care facilities in towns with populations below 20,000. Demographics, digital literacy, and attitudes towards AI were assessed. Statistical analyses included non-parametric tests and ordinal logistic regression. Most of the respondents expressed neutrality (43%) or a negative (31%) attitudes toward AI. Only 12.7% had direct experience with AI, and full trust in AI-assisted diagnoses was low (5.9%). Education was the strongest predictor of a positive AI attitude (*P* < 0.001); age was also significant (*P* = 0.04), while gender and place of residence were not. Most patients (86%) emphasized the importance of medical staff support.

Patients in areas of low digital literacy approach AI with cautious optimism, valuing its potential but requiring human oversight. To foster an equitable adoption of AI, communication and education efforts must address patient concerns and expectations.

## Introduction

Technological progress, particularly the dynamic development of AI-based solutions, is increasingly present and visible in the healthcare sector. Artificial intelligence, understood as a collection of advanced technologies, such as machine learning (ML), natural language processing (NLP), rule-based expert systems (RBES), robotics and robotic process automation (RPA), introduce new opportunities that allow advances in diagnostics, treatment, patient monitoring, and resource management of the healthcare system^1^. Predictive modeling helps detect early disease, while clinical decision support systems (CDSS) improve therapy precision, and the automation of administrative processes reduces the workload of healthcare professionals, as well as operating costs^4–9^.

Against the backdrop of mounting systemic challenges, such as aging societies, increasing demand for healthcare services, burnout experienced by healthcare professionals, and persisting staff shortages in the sector, AI-based solutions are not only reasonable but emerge as a pivotal resource for sustaining healthcare accessibility. According to data presented by Eurostat and the OECD, in Poland there are only 2.4 practicing physicians per 1,000 people, which situates the country at the very end of the EU scoreboard. The same situation holds true for nurses, 5.1 per 1000 people^10,11^. These shortages become especially acute in rural areas and smaller towns where access to healthcare services is often limited.

While AI holds the potential to improve service accessibility and quality, whilst also addressing critical issues of health equity and ethics^12^, successful implementation hinges on understanding societal attitudes and challenges^13^. Prior research highlights significant psychological barriers. Studies consistently show that physicians using AI are often perceived as less competent, trustworthy, and empathetic than those who do not^14^, highlighting the need for a trust-based framework for medical AI^5^. Even when medical advice is identical, patients rate it as less reliable if labelled as coming from „AI” or „Human + AI”. Crucially, physician supervision does not nullify this negative bias, and willingness to follow advice decreases if AI is involved^16^. This resistance is closely linked to complex psychological mechanisms^17^ and is frequently driven by „uniqueness neglect”—the fear that AI cannot account for a patient’s unique characteristics and symptoms^18^. Consequently, transparent communication and interventions that demystify algorithmic decision-making are vital for increasing acceptance^19^. However, a critical limitation of the existing literature is that most analyses of AI perception rely on surveys of Internet users, often via online questionnaires^20^. This approach inherently excludes individuals with limited digital literacy and Internet access—a significant demographic in Poland, particularly among the elderly and rural residents. According to the 2024 Polish Statistics report, only 48.8% of people aged 16–74 have basic digital skills, falling to just 13.7% in the 65–74 age group^21^. Furthermore, data from Statistics Poland (GUS) show that e-government usage is significantly lower among rural residents compared to urban dwellers (51.1% vs. 67.5%). This confirms that digital literacy varies significantly by residence and age.

With this in mind, the goal of the survey was to bridge the research gap by performing an analysis of the awareness, acceptance, and concerns surrounding the use of AI in healthcare among primary care patients in regions potentially endangered by digital exclusion — residents of rural areas and small towns in Poland. The study was also designed to identify demographic and psycho-social determinants of trust in AI and factors that condition the degree of acceptance of its use in healthcare settings.

## Results

### Demographic profile

The demographic profile of the study sample is presented in Table 1. The average age of the participants was 46 years and women prevailed in the sample (53.4%). Most respondents had secondary education (44.95%) and lived in the countryside (40.18%) or in small towns.

**Table 1:** Description of the study sample.

Variable	Value
Women; n (%) Men n (%) Non-binary persons No gender declared	291 (53.4) 239 (43.9) 3 (0.5) 12 (2.2)
Age (years); average ± SD, median (range)	45.92 ± 15.96 46 (18–86)
Place of residence: n (%) village city <10k 10-20k 20–50k 50-100k more than 100k not declared	219 (40.18) 143 (26.24) 155 (28.44) 11 (2.02) 6 (1.10) 5 (0.92) 6 (1.10)
Education: n (%) primary basic vocational secondary 1st degree studies (baccalaureate) 2nd degree studies (Master degree) not declared	24 (4.40) 95 (17.43) 245 (44.95) 89 (16.33) 86 (15.78) 6 (1.10)
Frequency of using primary care services: n (%) More often than once a month At least once a month At least once every quarter At least once every 6 months More rarely than once a year Not declared	15 (2.75) 71 (13.03) 189 (34.68) 159 (29.17) 110 (20.18) 1 (0.18)

## Self-assessment of digital literacy and experiences with e-health

**Fig. 1:**
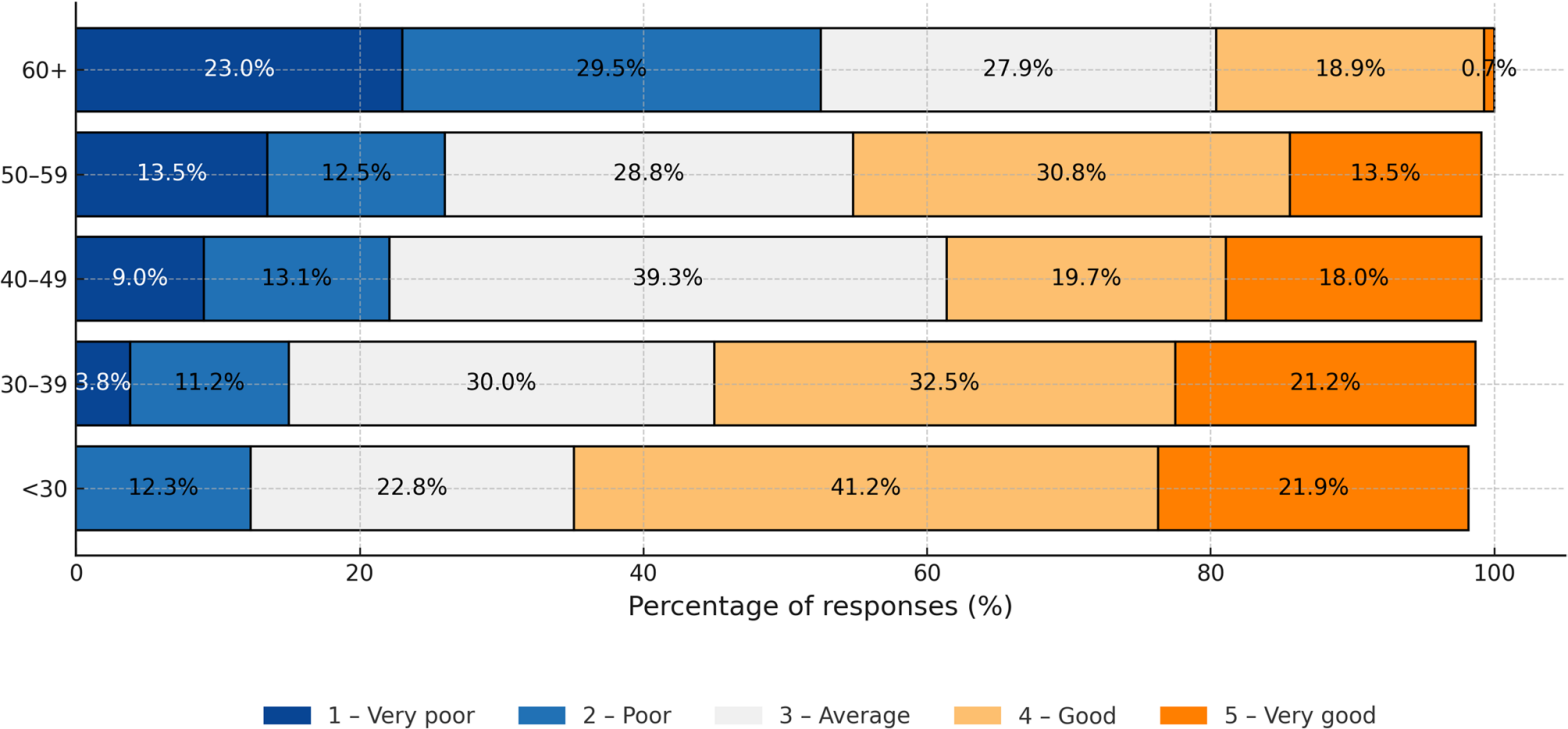
Self-assessed technology skills by age group.

**Fig. 2:**
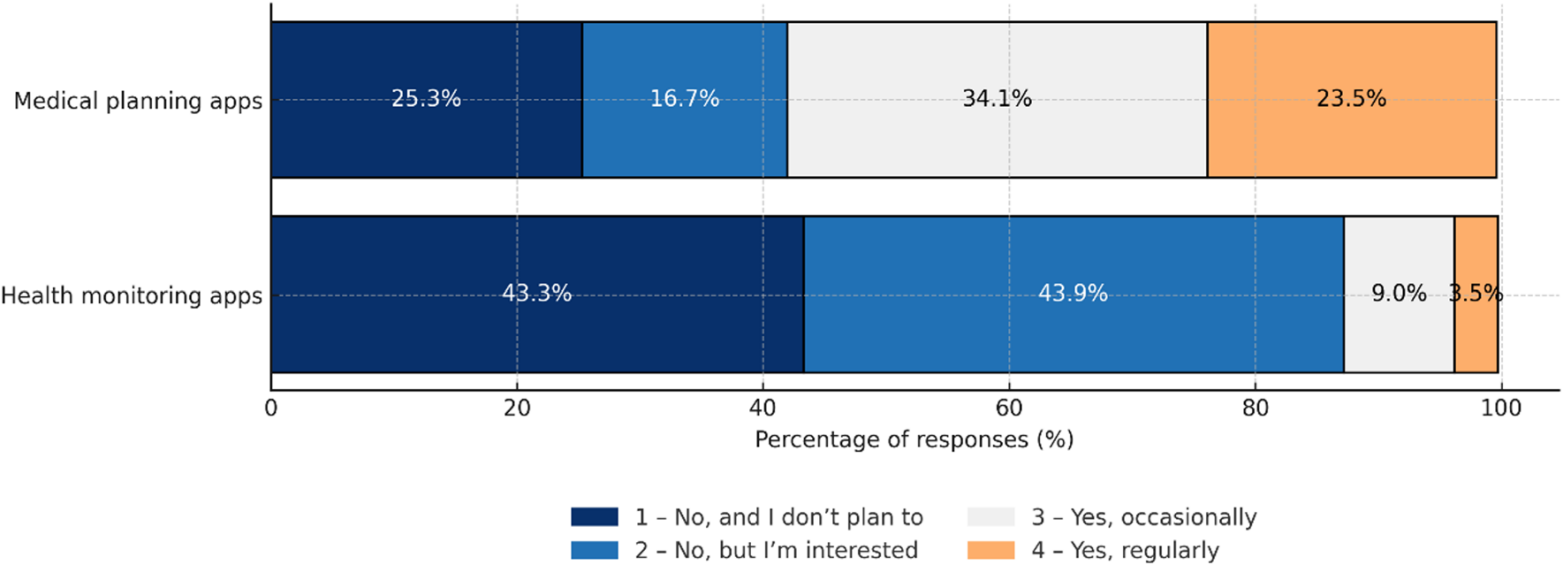
Use of medical and health monitoring applications.

- Almost 58% of respondents assess their digital skills as average or good, however, as many as 27% declare poor or very poor skills.
- A statistically significant negative correlation (rho = -0.35, *P* < 0.001) was observed between age and self-assessment of skills: the older the respondent, the lower self-assessment of skills (Fig. 1).
- More than half of respondents (57.8%) are familiar with applications used for planning healthcare services. Use of such applications also negatively correlated with age (rho = -0.28, *P* < 0.001). Health monitoring applications were much less popular, used either regularly or occasionally by only 12.5% of respondents (Fig. 2).

## AI awareness and attitudes in healthcare

**Fig. 3:**
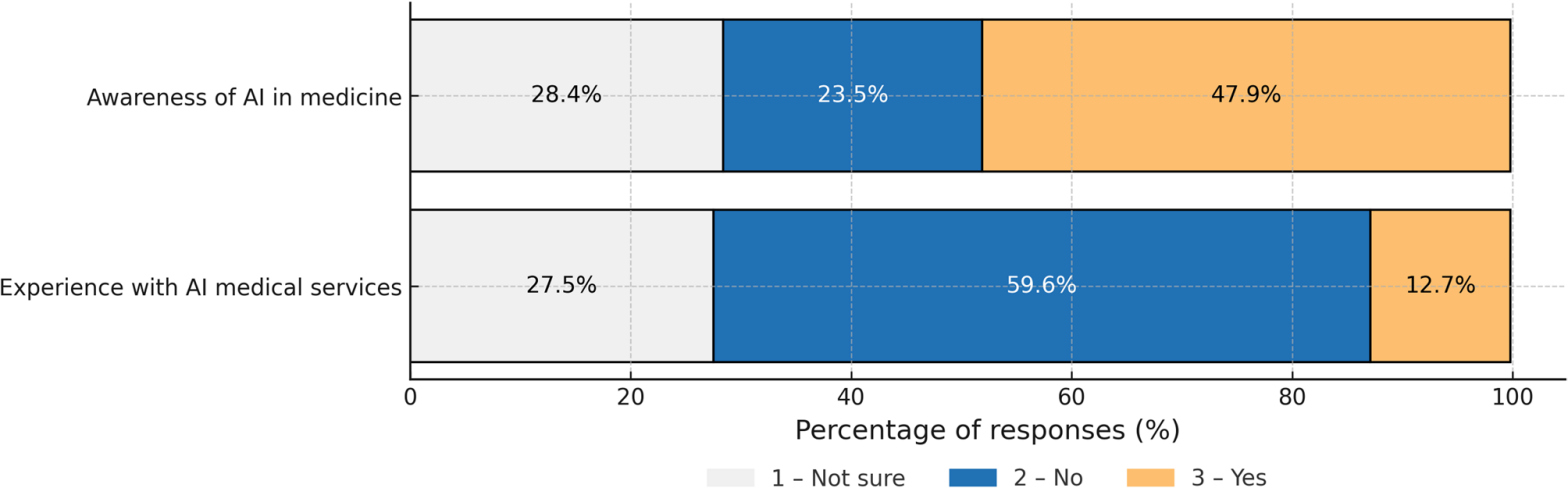
Awareness and experience with AI in healthcare.

**Fig. 4:**
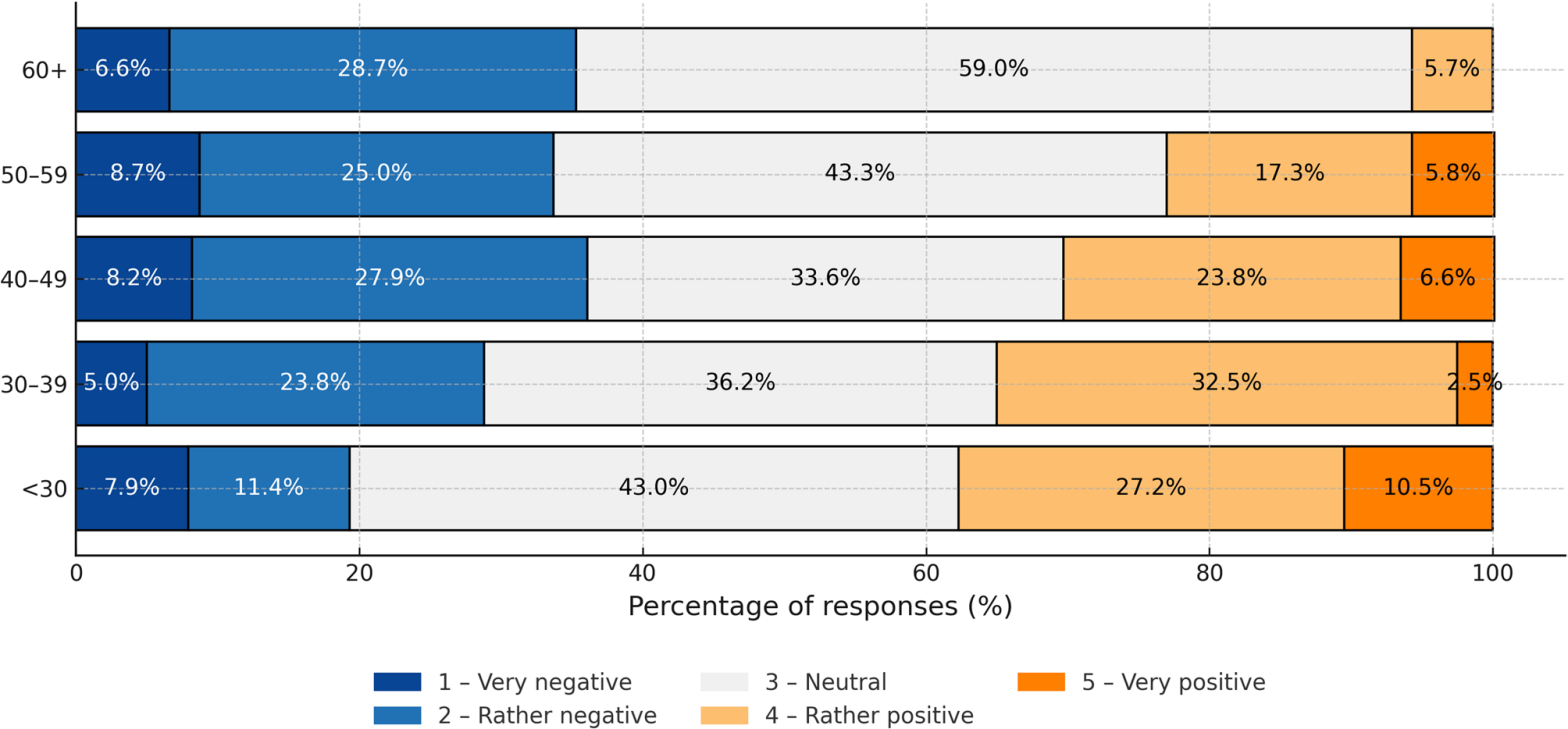
Attitudes towards AI in medicine by age group.

- Almost half of respondents (48%) are aware of AI applications in healthcare, but one quarter (24%) had never heard of them. Awareness was significantly poorer among elderly people. A mere 12.7% of respondents declared prior experience with AI-based healthcare service (Fig. 3).
- Attitude towards AI in healthcare was mainly neutral (43%). A positive attitude was declared by 25%, and a negative attitude by 31% of respondents. Younger age positively correlated with more positive attitude to AI (Fig. 4).

## Trust, the role of a physician, and concerns

**Fig. 5:**
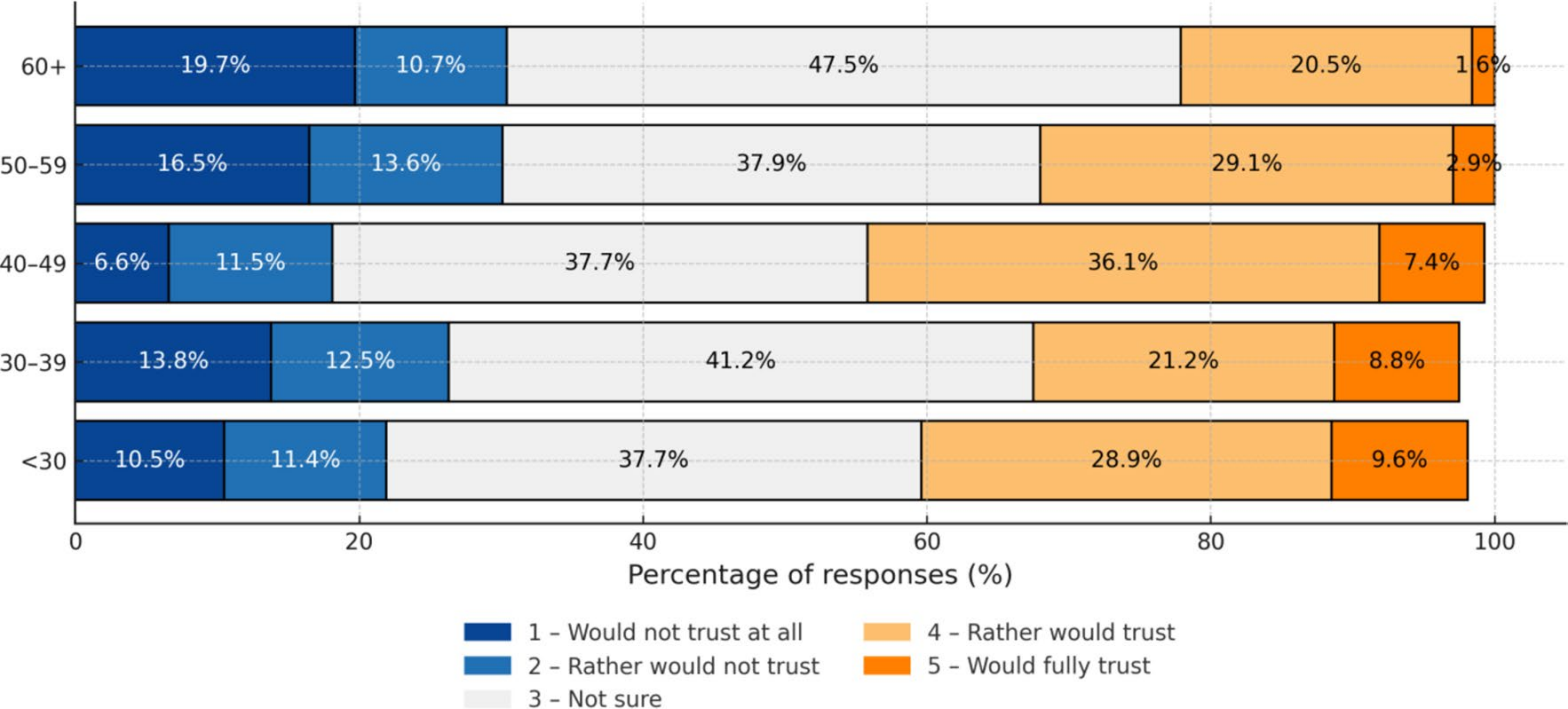
Trust in AI-assisted diagnosis supported by the doctor by age group.

**Fig. 6:**
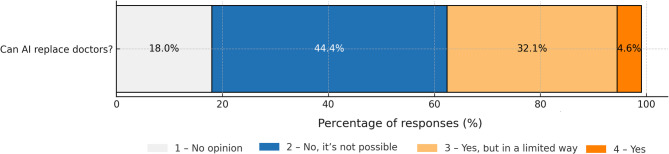
Opinions on whether AI can replace the doctor.

- Despite the neutral attitudes of the patients, trust in AI-assisted medical diagnosis is limited. As many as 40.7% of respondents are not sure whether they would trust an AI-made diagnosis, even if a physician were involved in the process. Full trust is declared by only 5.9% of patients (Fig. 5).
- Patients consider the physician to be irreplaceable. Almost 45% of respondents believe that AI cannot replace the doctor, and an additional 32.4% agree on engaging AI in the process only to a limited extent (Fig. 6).
- The main concern about AI reported was the lack of direct contact with the physician (197 responses) followed by the absence of a case-by-case approach, and the risk of errors.

## Expectations vis-a-vis AI-assisted solutions and AI future in healthcare

**Fig. 7:**
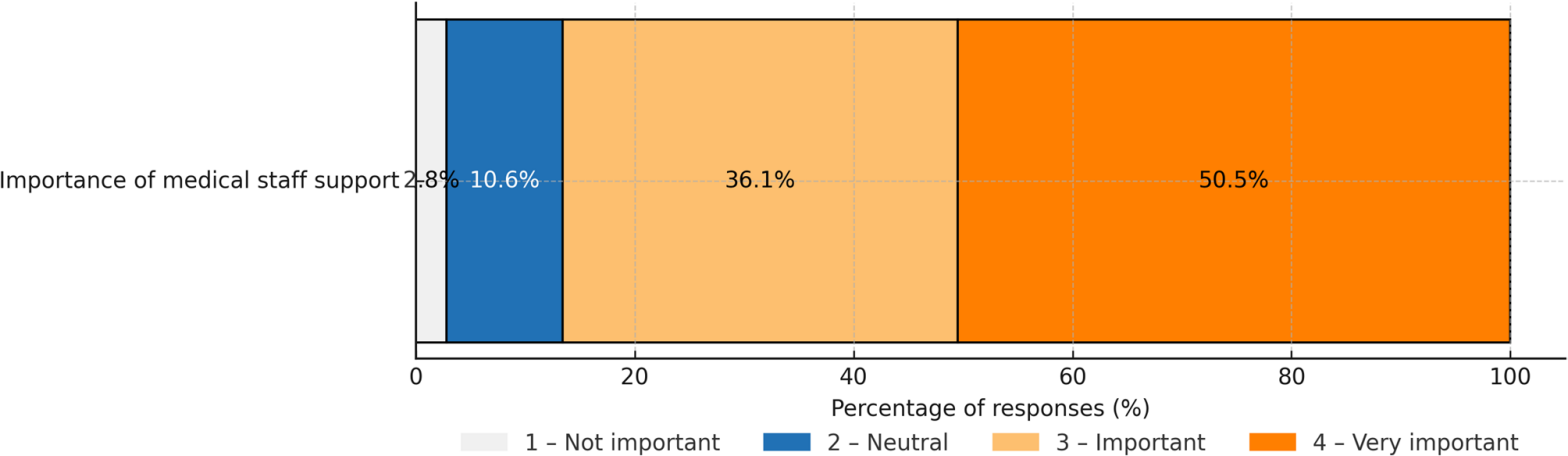
Importance of medical staff support when using AI.

**Fig. 8:**
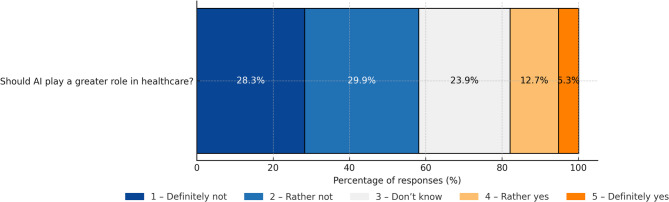
Opinions on AI playing a greater role in healthcare.

- More than 42% of respondents believe they lack the skills to use AI and would need some support. The availability of support from medical staff is seen as ‘important’ or ‘very important’ by more than 86% of respondents, and the need for such assistance increases with age (Fig. 7).
- Respondents are sceptical about AI-based future. Only 18% share the view that healthcare should pursue more AI-based solutions while 58% are against this path (Fig. 8).

By using a logistic regression model in statistical analysis, we obtained results that allowed us to prioritize the impact of the factors studied on attitudes towards AI.

### Predictors: education, age, place of residence, gender

The findings clearly demonstrated that education was the strongest predictor of patients’ attitudes toward artificial intelligence (OR = 1.92; 95% CI: 1.49–2.47; *P* < 0.001). This indicates that for each successive increase in education level, the chance of a more positive attitude increases by approximately 92%, assuming other factors remain constant. Age was also a statistically significant factor, though its impact was minor (OR = 0.98; 95% CI: 0.96–1.00; *P* = 0.04), indicating that each successive year of life slightly diminishes the likelihood of a positive attitude. The impact of the place of residence remained at the margin of statistical significance (OR = 1.10; 95% CI: 0.99–1.22; *P* = 0.08), suggesting a potential trend that warrants further investigation. Gender was not a significant predictor in this model (OR = 1.03; 95% CI: 0.75–1.41; *P* = 0.86).

## Discussion

This study revealed a significant awareness gap regarding AI in healthcare among the primary care patients in rural Poland. We found that just under half of the respondents (48%) had heard of AI applications, and only a small fraction (12.7%) reported any direct experience with an AI-based medical service. This level of awareness, while comparable to findings from Malta^22^, appears notably lower than that reported in other developed nations. For instance, studies report that general AI awareness exceeds 90% in Germany and is widespread in the United States; however, familiarity with specific healthcare applications remains less common in the latter^23,24^. Similarly, research in New Zealand indicated a higher awareness of AI in healthcare (58%) than observed in our cohort^25^. This disparity is likely attributable to two key factors. First, the lower overall digitization of the studied demographic — rural residents who are often older and less digitally native—naturally limits exposure to emerging technologies. Second, our use of paper-based questionnaires, a methodological choice to intentionally include this digitally excluded group, contrasts with the online surveys common in other studies, which intrinsically select for more digitally skilled respondents.

Beyond awareness, our study uncovered a nuanced and cautious attitude toward AI among the Polish rural patient population. While over two-thirds of respondents expressed neutral or positive views, a substantial minority (31%) held a distinctly negative stance. This cautious ambivalence contrasts with the more overtly positive attitudes reported in other highly digitized societies. For example, studies in Germany and New Zealand found high rates of positivity, with over 53% of Germans and 78% of New Zealanders viewing AI in medicine favorably^23,25^. While other research also describes a generally moderate or cautiously optimistic outlook^24,26^, the degree of negative sentiment in our cohort is noteworthy. We propose that this heightened caution is a direct reflection of the socio-cultural and technological context of our study group. Beyond awareness, our study uncovered a nuanced and cautious attitude toward AI among the Polish rural patient population. This cautious approach is consistent with the lower digital literacy observed in this demographic compared to participants in typical online surveys^21,27^. This aligns with broader findings that cultural context is pivotal in technology acceptance. For instance, studies in New Zealand have highlighted that indigenous Māori populations may prefer human physicians due to distinct cultural values, while research in France has shown that professional background shapes AI perceptions^25,28^. Therefore, the cautious stance observed in our study likely reflects not just a lack of familiarity, but also a deep-seated value placed on the human element in care within this specific community.

A striking finding of our study was the profound lack of trust in autonomous, AI-driven diagnoses. Only 6% of respondents expressed full trust in a diagnosis made solely by AI, and over 40% remained uncertain even if a physician were involved in the process. This finding stands in stark contrast to other research where patient trust in AI was reported to be significantly higher, in some cases comparable to trust in human doctors^25,29^. We argue that this apparent contradiction does not reflect a blanket rejection of AI, but rather a sophisticated, context-dependent assessment by patients. The discrepancy in trust levels across studies suggests that patients differentiate between various types of AI applications and their roles in the care pathway^30,31^. Trust appears to be highest for technologies that function as assistive tools in tasks where speed and accuracy are paramount, such as diagnostic imaging. Conversely, concerns escalate significantly for applications that encroach upon areas requiring empathy and holistic interpretation, as seen in fields like mental health^32^.

Stemming from this conditional trust is a near-universal demand for the physician’s irreplaceable role and ultimate accountability. In our study, a primary concern was the potential loss of human interaction, with respondents emphasizing that AI could only ever supplement, not replace, a physician. This sentiment is not unique to our cohort but is a consistent theme in international research. Studies from New Zealand to Germany echoed the same conclusion: patients expect AI to function as a support tool under the strict supervision of a clinician who remains responsible for the final diagnosis^23,25^. This places the burden of responsibility squarely on the healthcare professional, a view confirmed in studies where accountability for an incorrect AI diagnosis is assigned to the clinician^24^. Consequently, the successful and ethical integration of AI into healthcare hinges on developing clear regulatory frameworks that address fundamental questions of liability and data security^28^. In essence, the patient perspective strongly endorses a “human-in-the-loop” model, where technology enhances clinical capabilities, but the physician’s expertise and ethical accountability remain central.

Our analysis of demographic predictors confirms that education and age are pivotal in shaping patient attitudes, a finding consistent with international literature^22–26^. Higher educational attainment correlated with greater acceptance of AI, while older age was associated with more skepticism, likely linked to lower self-assessed digital literacy^23,24,33^. Intriguingly, some studies suggest this relationship is not linear, as younger, more digitally-aware patients can also be more critical of AI, perhaps due to a greater understanding of its limitations and ethical risks^25^. In our cohort, gender did not emerge as a significant predictor of attitude, which contrasts with some research suggesting women may adopt new technologies more cautiously^23,26^.

The study’s primary strength lies in its focus on a medically and digitally underserved population, using paper-based methods to bridge a critical gap in research that often relies on digitally native, online respondents. However, several limitations should be acknowledged. First, the use of purposive sampling limits the generalizability of our findings to the broader patient population in Poland. Second, our regression model did not include all potential confounding variables that might influence patient attitudes, such as prior experiences with the healthcare system. Third, the use of a novel, non-standardized survey instrument, though piloted, may affect direct comparability with studies using validated scales. Finally, the interpretation of these findings must account for the inherent limitations of survey methodology, including social desirability bias and demand effects. While surveys provide a broad overview of stated attitudes, they may not fully capture the complexity of behavioral responses in real-world scenarios. Recent experimental studies suggest that patients often exhibit more negative attitudes toward medical AI in practice than self-reported survey data might imply^16^. Consequently, the acceptance levels reported here should be viewed as a baseline for stated preferences rather than a definitive predictor of clinical behavior. Future research could build upon our findings using mixed-methods to explore these cultural and experiential nuances in greater depth.

Our findings offer practical implications for stakeholders navigating the digital transformation of healthcare. First, there is a clear need for targeted education and transparent communication campaigns to explain the function, benefits, and limitations of AI, particularly for older patients and those with lower digital literacy. Second, AI systems must be designed with the user in mind, featuring intuitive interfaces and easily accessible human support channels. Most critically, any implementation strategy must unambiguously communicate that AI is a supportive tool and that the final clinical decision remains with the human physician. This “human-in-the-loop” guarantee is a non-negotiable prerequisite for building the social trust necessary for successful adoption.

In conclusion, patients from this underserved community approach AI in healthcare with cautious but pragmatic optimism. They recognize its potential to enhance diagnostics, but only under the condition of strict physician supervision and accountability. Their primary concerns are not purely technical but are deeply rooted in the humanistic aspects of care: namely, the paramount importance of preserving the empathetic, personalized patient-doctor relationship. These findings underscore a critical mandate for the future of digital medicine. Successful AI integration cannot be solely a technological challenge; it must be a human-centric endeavor that respects patient values, guarantees clinical oversight, and purposefully designs technology to augment, rather than replace, the irreplaceable human dimension of healthcare.

## Methods

### Study design and sample selection

A multicenter cross-sectional study was conducted from December 1 to December 31, 2024. The study design involved collecting data at a single point in time to assess the attitudes, knowledge, and concerns of primary care patients regarding the use of artificial intelligence (AI) in healthcare.

The selection of research sites was purposeful. The key selection criterion was the location of the Primary Health Care (PHC) center in a rural area or in a city with a population of less than 20,000. The rationale for this approach is the demographic structure of Poland, where a significant proportion of citizens (according to statistical data, over 60%) live in such areas^27^. The aim was to deliberately reach the perspective of this often-underrepresented group in research on new technologies in medicine, which increases the external validity and generalizability of the results to the wider Polish population^34,35^. Ultimately, three PHC centers located in central Poland meeting the above criteria were selected for the study.

Purposive sampling was used, based on patient availability. Interviewers invited all consecutive adults present in the waiting rooms of designated facilities on specific days and times to participate in the study. The criteria for inclusion in the study were: age over 18, patient status at the given facility, and voluntary informed consent. The exclusion criteria were the inability to read and complete the questionnaire independently (e.g., due to cognitive impairment or language barriers) and refusal to participate. Participation in the study was completely voluntary and anonymous, and patients were informed that refusal would not affect the quality of their current or future medical care. Of the 600 patients invited, 545 agreed to participate and correctly completed the questionnaire, resulting in a response rate of 90.8%.

### Research tool

The research tool was a proprietary, anonymous paper-based survey questionnaire. It was developed based on a comprehensive review of the relevant literature in PubMed, Scopus, and Web of Science databases, using the following keywords: “artificial intelligence,” “healthcare,” “patient acceptance,” “technology acceptance model,” and “UTAUT.” The design of the questionnaire was inspired by the assumptions of the Technology Acceptance Model (TAM) and the Unified Theory of Acceptance and Use of Technology (UTAUT), adapting their key constructs to the specific context of AI in healthcare.

The questionnaire consisted of four parts: introduction, socio-demographic data, self-assessment of digital knowledge and competence, and attitudes towards AI in healthcare.

The introduction contained detailed information about the purpose of the study, its voluntary and anonymous nature, the institution conducting it, and contact details for the researchers. The approval of the bioethics committee was also emphasized.

The socio-demographic data included questions about gender, age (in the following ranges: 18–34, 35–49, 50–64, ≥ 65 years), level of education (primary/vocational, secondary, higher), and place of residence (rural, urban < 20,000).

The section on self-assessed digital literacy included questions aimed at determining patients’ general knowledge of AI and their ability to use digital technologies (e.g., smartphones, health apps).

Attitudes towards AI in healthcare: The main part of the questionnaire contained questions about patients’ attitudes towards AI in healthcare. The questions concerned perceived benefits (e.g., faster diagnosis), potential barriers and risks (e.g., loss of empathy, data security), and trust in AI systems.

Before the actual study began, a pilot study was conducted on a group of 20 primary care patients with diverse demographics. The purpose of the pilot study was to verify the comprehensibility, clarity, and logical consistency of the questions and to estimate the average time needed to complete the survey (approximately 10 min). Based on the feedback received, minor editorial corrections were made to three questions to improve their clarity. The English version of the questionnaire used in this study is available from the corresponding author upon request.

### Statistical analysis

The data collected from paper surveys were digitized by double-entry into a spreadsheet to minimize the risk of errors. Statistical analyses were performed using the Python programming language (version 3.12). Data processing and analysis relied on open-source libraries: pandas (for data management), scipy.stats (for statistical tests), and statsmodels (for regression modeling).

The normality of the distribution for continuous and ordinal variables was assessed using the Shapiro-Wilk test, which showed that the key variables were not normally distributed (*p* < 0.05). Therefore, appropriate non-parametric tests were used for further analysis.

Spearman’s rank correlation coefficient (ρ) was used to assess the strength and direction of the relationship between ordinal variables (e.g., level of education and attitudes toward AI). The Mann-Whitney U test was used to compare the distributions of the dependent variable in two independent groups (e.g., comparison of attitudes between males and female).

The main analytical tool employed was the proportional odds model in ordinal logistic regression. The dependent (explained) variable was the overall attitude of patients towards AI, categorized on a 5-point scale (1 – “very negative,” 2 – “negative,” 3 – “neutral,” 4 – “positive,” 5 – “very positive”). The independent variables (predictors) in the model were: age, gender, level of education, and place of residence of patients. The regression results were presented as odds ratios (OR) with 95% confidence intervals (CI). In all statistical analyses, a priori significance level of α = 0.05 was adopted. To account for multiple comparisons in the correlation and difference analyses (Tables 2 and 3), we applied the Benjamini-Hochberg procedure to control the False Discovery Rate (FDR). In cases where the p-value obtained was extremely low, it was reported as *P* < 0.001.

**Table 2:** Spearman’s Rho results in the correlation between the respondent’s educational background and her/his responses.

Analysed aspects	Education	
	Spearman’s Rho	*P*
Frequency of using GP services	-0.16	< 0.001*
Self-assessment of skills in the field of modern technologies	0.61	< 0.001*
Use of applications that streamline planning of healthcare services	0.40	< 0.001*
Use of health monitoring applications	0.37	< 0.001*
Awareness of using AI in medical contexts	0.36	< 0.001*
Direct experience with AI-based healthcare services	0.052	0.23
Attitude towards using AI in medicine	0.38	< 0.001*
Trust in AI diagnosis if made with the involvement of the doctor	0.37	< 0.001*
Concerns about the physician being replaced with AI in healthcare	0.19	< 0.001*
Belief that AI can improve the quality of healthcare	0.32	< 0.001*
Self-assessment of technological skills needed to use AI-based solutions in healthcare	0.46	< 0.001*
Readiness to learn about AI applications in medicine	0.49	< 0.001*
Relevance of support offered by the medical staff when using AI-based systems in medical contexts	-0.07	0.09
Patients’ belief that the future of healthcare should rely much more on AI	0.4	< 0.001*

* Indicates statistical significance after applying the Benjamini-Hochberg correction for multiple comparisons (FDR < 0.05). Raw P-values are reported.

**Table 3:** Results of Mann-Whitney tests – analysis of responses broken down by gender (women vs. men) and place of residence (city vs. country).

Women vs. men		Analysed aspects	City vs. country	
*r* effect size	*P*		*r* effect size	*P*
0.159	< 0.001*	Frequency of using GP services	0.127	0.002*
0.075	0.08	Self-assessment of skills in the field of modern technologies	0.252	< 0.001*
0.127	0.002*	Use of applications that streamline planning of healthcare services	0.294	< 0.001*
0.007	0.85	Use of health monitoring applications	0.222	< 0.001*
0.03	0.46	Awareness of using AI in medical contexts	0.236	< 0.001*
0.045	0.24	Direct experience with AI-based healthcare services	0.063	0.09
0.069	0.09	Attitude towards using AI in medicine	0.141	< 0.001*
0.058	0.16	Trust in AI diagnosis if made with the involvement of the doctor	0.172	< 0.001*
0.013	0.75	Concerns about the physician being replaced with AI in healthcare	0.045	0.26
0.045	0.28	Belief that AI can improve the quality of healthcare	0.046	0.27
0.031	0.44	Self-assessment of technological skills needed to use AI-based solutions in healthcare	0.143	< 0.001*
0.003	0.95	Readiness to learn about AI applications in medicine	0.188	< 0.001*
0.076	0.05	Relevance of support offered by the medical staff when using AI-based systems in medical contexts	0.034	0.38
0.042	0.31	Patients’ belief that the future of healthcare should rely much more on AI	0.044	0.29

* Indicates statistical significance after applying the Benjamini-Hochberg correction for multiple comparisons (FDR < 0.05). Raw P-values are reported.

### Ethical principles

The study was designed and conducted in accordance with the principles of the Declaration of Helsinki. The experimental protocol was approved by Independent Bioethics Committee operating at the Medical University of Lodz (consent no. RNN/230/24/KE of November 19, 2024).

Each potential participant was informed about the study and asked to give their informed consent. Full anonymity was ensured by not collecting any personally identifiable information (first names, last names, Polish national identification numbers). The completed questionnaires were stored in a secure location, and the digitized data were stored on an encrypted disk accessible only to the research team. Data processing was conducted in accordance with applicable data protection regulations, including Regulation (EU) 2016/679 of the European Parliament and of the Council (GDPR).

## Data Availability

Due to the ongoing nature of the research, the current dataset is not publicly available. To access the data, please contact the corresponding author by e-mail.
